# Beneficial Effect of Antibiotics and Microbial Metabolites on Expanded Vδ2Vγ9 T Cells in Hepatocellular Carcinoma Immunotherapy

**DOI:** 10.3389/fimmu.2020.01380

**Published:** 2020-07-22

**Authors:** Jiajia Han, Siya Zhang, Yi Xu, Yongsheng Pang, Xue Zhang, Yu Hu, Hui Chen, Wanjun Chen, Jianmin Zhang, Wei He

**Affiliations:** ^1^CAMS Key Laboratory for T Cell and Immunotherapy, State Key Laboratory of Medical Molecular Biology, Department of Immunology, Institute of Basic Medical Sciences, Chinese Academy of Medical Sciences and School of Basic Medicine, Peking Union Medical College, Beijing, China; ^2^Mucosal Immunology Section, National Institute for Dental and Craniofacial Research, National Institutes of Health, Bethesda, MD, United States

**Keywords:** γδT cells, tumor immunotherapy, antibiotics, 3-indolepropionic acid, microbiota

## Abstract

Animal experiments and clinical trials have shown that the gut microbiota modulates host immunity and immune checkpoint-mediated responses to tumor cells. However, it remains unclear whether microbiota can also play a role in the tumor immune response of γδT cells, a kind of cell that targets cancer directly. Here, we report that microbiota dysbiosis induced by antibiotics enhanced γδT cell efficacy during tumor therapy in a mouse model. Further microbiota and metabolite analysis revealed that the alteration of γδT cell cytotoxicity might be closely associated with specific metabolites, which are produced by intestinal bacteria and stimulate γδT cells to release more cytotoxic cytokines, such as granzyme B and perforin. Among the metabolites that we analyzed, 3-indopropionic acid (IPA) showed the highest concentration in antibiotic-treated mice and can improve the cytotoxic ability of γδT cells both *in vitro* and *in vivo*. Our research determined how the gut microbiota can influence the antitumor ability of γδT cells and identified potential intermediate molecules that connect the gut microbiota and γδT cells.

## Introduction

γδT cells arise from double-negative precursors in the thymus, and their T cell receptor (TCR) is characterized by γ and δ chains that are generated via somatic recombination (1). The Vγ and Vδ gene segments are extremely limited in γδT cells, and according to the usage of these segments, human γδT cells can be divided into three major subsets and have distinct distribution preferences in terms of different tissues (2). Vδ1 is dominantly distributed in the skin and mucous membranes (3), Vδ3 in the liver, and Vδ2 in the peripheral blood (4). Although <10% in peripheral T cells, Vδ2 Vγ9 cells can be easily expanded *in vitro* and applied for clinical immunotherapy based on the characteristics that can be activated by phosphoantigens (5).

It is already known that Vδ2Vγ9 cells not only can recognize antigens by complementarity determining region 3 (CDR3) in a major histocompatibility complex (MHC) unrestricted manner, thus responding to tumor cells directly, but also have a wide antigen recognition spectrum, including soluble proteins, smaller peptides, prenyl pyrophosphates, phospholipids, and sulfolipids (6–8). NKG2D is one of the most important receptors expressed on Vδ2Vγ9 T cell membrane, which increases the interaction of ligand manifold to it. In patients with Epstein–Barr virus-induced lymphoproliferative disease (EBV-LPD) after transplantation, expanded Vγ9Vδ2 T cells enable the destruction of autologous lymphoblastoid B cells in a γδ TCR- and NKG2D-dependent manner (9). MHC class I polypeptide-related sequences A and B (MICA/B) and some stress-related proteins, such as DNA mismatch repair protein MutS homolog 2 (MSH2), UL16-binding protein 1 (ULBP1), ULBP2, ULBP3, ULBP4, ULBP5, ULBP7, and ULBP9, are highly expressed under stress and can be targeted by Vδ2 Vγ9 T cells.

These specificities indicate that γδT cells can recognize more diverse tumor antigens than αβT cells, and some studies also found that γδT cells can infiltrate inside B cell lymphomas, prostate cancer, breast cancer, melanoma, acute myeloid leukemia (AML), gastric cancer, neuroblastoma, pancreatic adenocarcinoma, colon cancer, and so on (10–12). Furthermore, Vδ2Vγ9 T cells possess the characteristics of self-activation and release the Th1-type cytokine interferon gamma (IFN-γ) and other cytotoxic cytokines, such as tumor necrosis factor (TNF), perforin, and granzymes (granzyme A and B), to eliminate tumor cells (8, 13, 14). These Vδ2Vγ9 T cells can also recognize upregulated isopentenyl pyrophosphate (IPP) and mevalonate pathway intermediates expressed on tumor cells, thus against the mutated cells by cytotoxic effect rather than the normal cells. These advantages assist Vδ2Vγ9 T cells in efficiently and precisely interacting and destroying cancer cells and make these cells a promising treatment for curing tumors, especially strategies based on expanded cells by zoledronate or anti-TCR pans with IL-2 from human peripheral blood mononuclear cells (PBMCs) (15). Moreover, vitamin C (l-ascorbic acid) is another promising strategy to improve γδT cell efficacy in tumor therapy by promoting proliferation and effective function (16).

Autologous and allogeneic Vδ2Vγ9 T cell adoptive immunotherapies are two ways widely used to apply γδT cells for clinical patients. Some clinical trials have shown the evidence of Vδ2^+^ T cell response to various tumors, especially for hematological malignancies, such as non-Hodgkin's lymphoma and acute myeloid leukemia, as well as for some solid tumors, such as prostate cancer, breast cancer, colon cancer, and ovarian cancer. Although Vδ2Vγ9 T cell adoptive immunotherapy gains success in people with different diseases, not all patients respond to this strategy. On the other hand, the efficacy of γδT cell immunotherapy for human cancer is usually not as good as we expected in theory for unknown reasons. In general, <30% of tumor patients respond to γδT cell immunotherapy, but even when tumors are specifically targeted by γδT cells, ~30% of patients achieve stable disease rather than partial or complete cure (17–19). These facts indicate that more effort needs to be made to improve the cytotoxicity of γδT cell immunotherapy. Therefore, we tried to determine what kind of factors *in vivo* may influence the cytotoxicity of γδT cells in tumor immunotherapy.

According to recent studies, the gut microbiota regulates the activities of multiple systems and has an intimate connection with the immune system (20, 21). There is evidence that the microbiota enables the modulation of immunotherapy of CD8+ T cells against tumors via TLR4, as well as anti-PD-1 immunotherapy, by downregulating the ratio of effector T cells and regulatory T cells (22–24). As a powerful treatment for the disordered gut microbiota, antibiotics have also been shown to inhibit the benefit of immune checkpoint inhibitor therapy for cancer in patients. Therefore, we are interested in whether the microbiota can also play a role in γδT cell immunotherapy for cancer. To understand this, we used HepG-2 human hepatocellular carcinoma-bearing nude mice, given expanded human Vδ2Vγ9 T cell therapy with or without antibiotics, and then measured the size of the tumor. Additionally, we also examined the profile of the gut microbiota and its related metabolites to further explore how the microbiota regulates γδT cell cytotoxicity. From these results, we found that the microbiota is a factor *in vivo* that modulates the cytotoxicity of γδT cells during tumor immunotherapy, and the metabolite produced by gut microbiota is the key mediator connecting γδT cells and gut microbiota.

## Methods and Materials

### Flow Cytometry and Antibodies

Antihuman FITC-TCR γδ, antihuman APC-TCR γδ, antihuman PE-TCR γδ, antihuman FITC-Vδ1, antihuman PercpCy5.5-Vδ2, antimouse FITC-TCRγδ, antimouse APC-TCRγδ, antimouse PercpCy5.5-CD3, antihuman PE-Vγ1.1, antihuman APC-Vγ1.2, antimouse PE-CD11c, antimouse PercpCy5.5-NK1.1, antimouse APC-CD19, antimouse PE-TLR4, antimouse FITC-NKG2D, antimouse PercpCy5.5-IFN-γ, and antimouse PercpCy5.5-IL17a antibodies were obtained from Biolegend and eBioscience. Cytokine staining was examined with an eBioscience™ Foxp3/Transcription Factor Staining Buffer Set (Thermo Fisher).

### Animal Models

Eight-week-old adult C57BL/6J black mice and 4- to 6-week-old BALB/c nude mice were housed in the Experimental Animal Center of Peking Union Medical College under specific-pathogen-free (SPF) conditions. All the procedures in this study were authorized and supervised by the Animal Care and Use Committee of Peking Union Medical College. All procedures were executed in safe circumstances and strictly followed the biosecurity rule in Peking Union Medical College.

### Cell Lines and Culture

HepG-2 human hepatocellular carcinoma cells were purchased from American Type Culture Collection (ATCC) and transfected with luciferase-GFP lentivirus, and the selected pure GFP-labeled cells were sorted with a Moflo-XDP high-speed flow cell sorter and cultured with Eagle's minimum essential medium (EMEM) supplemented with 10% fetal bovine serum. Human NCI-H520 and NCI-H446 cells were purchased from ATCC and cultured with Roswell Park Memorial Institute (RPMI)-1640 supplemented with 10% fetal bovine serum.

### IEL Isolation

Small intestine was isolated from mice, Peyer's patches and fat attached to the tissue were removed, and the intestine was cut open. Feces and mucus were washed away with phosphate-buffered saline (PBS), and the gut tissue was cut into pieces, suspended in RPMI-1640 plus 5% fetal bovine serum and placed on a shaker for 20 min (37°C, 180 rpm). Then, the samples were centrifuged at 300 *g* for 10 min, the cells were suspended in 44% Percoll, and 70% Percoll was added slowly from the bottom. The samples were centrifuged at 1,800 rpm for 20 min with brake 0, and lymphocytes were obtained between these two layers (25).

### Human PBMC Isolation and γδT Cell Expansion

Fresh human PBMCs were separated from peripheral blood from healthy donors by density gradient centrifugation on Ficoll-Hypaque (Pharmacia) and cultured using RPMI-1640 (Gibco BRL) medium with 10% fetal calf serum (FCS) and IL-2 (200 U/ml) in 24-well culture plates coated with an anti-TCR pan-γδ antibody (2 ng/ml) at a density of 2 × 10^6^ cells per well to expand pure γδT cells (15). For γδT cell frequencies and proliferation examination stimulated by fecal extract, γδT cells were incubated in wells coated with anti-TCR pan-γδ (10 ng) for 2 h at 37°C (positive control) or treated with fecal dilutions (20 μl) from different mice or an equivalent volume of PBS. PBMCs from peripheral blood from the same healthy donor were cultured in 24-well plates at 2 × 10^6^ cells per well using RPMI-1640 complete medium for 5 days and then detected by flow cytometry.

### Tumor Incubation and Therapy Options

The concentration of hepatic carcinoma HepG2 cells was adjusted to 4 × 10^7^/ml, and 50 μl of the suspension was injected under the back skin of each BALB/c nude mouse. When the tumor diameter grew to 5–9 mm, the mice were randomly divided into four groups: control (Ctrl), antibiotic in the drinking water (ATB), γδT cell therapy (γδT), and antibiotic in the water combined with γδT cell therapy (ATB + γδT). A total of 1 × 10^7^ γδT cells in a volume of 80 μl containing 5,000 IU IL-2 were injected intravenously into each mouse, and γδT cell treatment was performed five times at an interval of 4 days.

### Antibiotic Treatment

C57BL/6J mice were given drinking water containing antibiotics (penicillin, 0.8 mg/ml; chloramphenicol, 1 mg/ml; streptomycin, 1.2 mg/ml) (24, 26) 1 week before harvest. Tumor-bearing nude mice were given drinking water containing antibiotics the day before γδT cell therapy and 4 days later, followed by normal water after 4 days, and this cycle was repeated until the end of therapy.

### 16S rRNA Gene Sequencing

DNA was extracted from fecal samples, and the concentration was detected using the Qubit® dsDNA HS Assay Kit. Then, the highly variable region (V3 and V4) of 16S rDNA was amplified with primers designed by GENEWIZ. Concentrations were then measured with a Qubit3.0 Fluorometer (Invitrogen, Carlsbad, CA) and adjusted to 10 nM for PE250/FE300 double-end sequencing. Finally, data analysis was performed.

### Fecal Collection and Sterile Suspension Preparation

Feces (240 mg) were suspended in 3 ml PBS and centrifuged at 5,000 rpm for 5 min at 4°C. The supernatant was collected and filtered with a 0.22-μm filter screen, and the final product was stored at −80°C.

### *In vitro* Cytotoxicity Testing of γδT Cells by CytoTox 96® Non-radioactive Cytotoxicity Assay

The CytoTox 96® Non-radioactive Cytotoxicity Assay kit was purchased from Promega (United States, G1780). Expanded γδT cells were cultured *in vitro* in 96-well plates with tumor cells at a 10:1 ratio (1 × 10^4^ tumor cells and 10^5^ γδT cells in each well). Then, 10 μl of PBS, filtered fecal suspension, or different doses of 3-indopropionic acid (IPA) was added, and the plate was centrifuged for 4 min at 250 *g* and incubated at 37°C for 4 h. After centrifugation, 50 μl of the supernatant was transferred to a new 96-well plate, 50 μl of reaction substrate was added, and the plate was incubated for 30 min at room temperature away from light. Then, 50 μl of stop buffer was added, and the optical density (OD) value was measured to calculate the cytotoxicity of γδT cells according to a formula.

### ELISA Detection of Cytokines

Expanded γδT cells were cultured to day 9, and the purity of γδT cells was more than 90%. The cells were collected and counted and then transferred into 48-well plates with 1 × 10^6^ cells per well. Sterile diluents of mouse feces were added to each well in a volume of 30 μl. After 24 h, the cells were collected, and the supernatant was obtained after centrifugation at 300 g for 10 min. The supernatant was diluted three times with sample diluent, added into coated plates, and incubated at 37°C for 2 h. Then, antibodies labeled with biotin were added and incubated at 37°C for 1 h. Next, horseradish peroxidase (37°C for 1 h) was added, and the substrate solution (37°C half an hour) was added. Then, the reaction was stopped with stop solution, and the OD value was measured.

### Metabolite Detection by Gas Chromatography/Mass Spectrometry

Fecal feces and tumor tissues were collected from mice and stored at −80°C until gas chromatography coupled to time-of-flight mass spectrometry (GC-TOFMS) analysis. Sample preparation was based on articles published by Metabo-Profile Biotechnology Co., Ltd. (Shanghai) (27). Gas chromatography coupled to time-of-flight mass spectrometry was used to quantitate the microbial metabolites, and then analysis, as well as normalization, quantitation, and statistics, was performed by XploreMET software (v2.0, Metabo-Profile, Shanghai, China).

## Results

Antibiotics, medicines that are widely used in the clinic to prevent infection, elicit an imbalance of the normal intestinal microbiota by eliminating many bacteria that are sensitive to them. To diminish the majority of bacteria in the gut, we chose three classical antibiotics (penicillin, chloramphenicol, and streptomycin) as antibiotic treatments. We gave C57BL/6 mice drinking water containing antibiotics and tested their microbiota distribution in the gut. It is not surprising that the general microbial diversity and abundance of these mice are extremely reduced (Supplementary Figures 1C–F). Moreover, the cecum of ATB-treated mice was obviously larger and had more content than that of the control mice (Supplementary Figures 1A,B). To further explore whether antibiotic treatment can also influence peripheral immune cells, we examined γδT cells, B cells, natural killer cells, and dendritic cells in the spleen (Supplementary Figures 2A–D) and mesenteric lymph nodes (Supplementary Figures 2E,F) of these mice; although the frequency of B cells and dendritic cells was not significantly different between these two groups, γδT cells in the spleen and NK cells in the mesenteric lymph nodes were decreased in antibiotic-treated mice. We also found that ATB-treated mice have more intraepithelial lymphocyte (IEL) γδT cells; both the frequency and total number of Vγ1 and Vγ4 are increased (Supplementary Figures 3A–C), but the Vγ1^−^Vγ4^−^ subset (mostly Vγ7) is not significantly different (Supplementary Figure 3D). We examined cytokines and surface markers related to antimicrobials that can be secreted or expressed by γδT cells. γδT cell in ATB-treated mice secrete more IL-17 and IFN-γ and express higher TLR4 but not NKG2D (Supplementary Figure 3E).

### Antibiotic Treatment Promotes γδT Cell Immunotherapy Against Tumors

**Figure 1:**
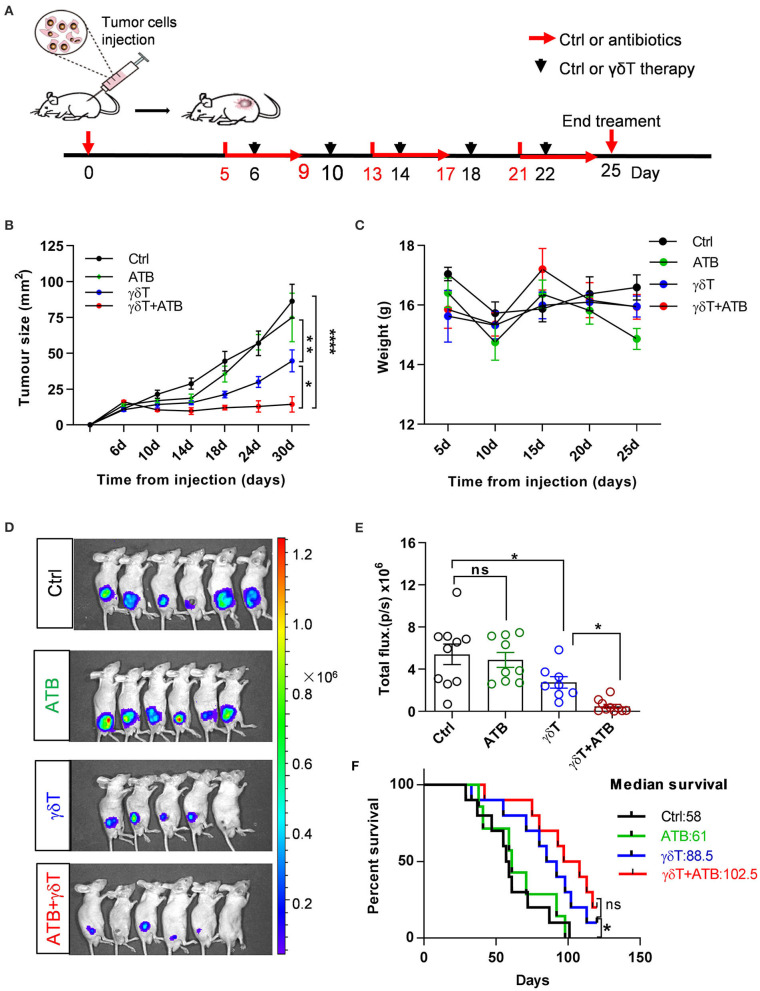
Antibiotic treatment promotes γδT cell immunotherapy for tumors. **(A)** Experimental design and time point of treatment. **(B)** Curves of tumor growth for 8–18 mice in each group and represent the results of three independent experiments. Analysis of variance (ANOVA), *p < 0.05, **p < 0.01, and ****p < 0.0001. **(C)** Mouse weight during treatments in these groups. **(D)** Detection of tumor growth via bioluminescence imaging of luciferase at day 25. **(E)** Statistical graph of total fluorescence intensity from bioluminescence imaging of luciferase, including three independent experiments. Each group had 8–10 mice. Student's *t*-test. *p < 0.05 and ns, no significant. **(F)** Survival curves of tumor-bearing mice. Each line represents one survival curve for each group of four or five mice from two or three independent experiments. log-rank (Mantel–Cox) analysis and *p < 0.05, ns, no significant. Data are the means ± SEM.

Based on the results of previous experiments, we conclude that alterations in the proportion, composition, and phenotype of peripheral immune cells are closely related to the distribution of the intestinal flora in mice. Antibiotics are usually widely used in patients with immunotherapy, but whether this intake can modulate the healing efficacy of γδT cells against tumors needs to be discussed. Therefore, we focused on this question in the next study. To plant human HepG2-Luciferase hepatocellular carcinoma cells successfully, BALB/c nude mice were inoculated with tumor cells subcutaneously on the right side of the back and then randomly divided into several groups and given different treatments when the tumor diameter reached 5–9 mm (Figure 1A). The purity and composition of expanded human γδT cells are shown in Supplementary Figure 4. After five rounds of therapy, the tumor size in mice with γδT cell therapy and γδT cells combined with antibiotic therapy (γδT + ATB) was dramatically decreased compared to that of the other two groups (ATB and blank, *p* < 0.05; Figure 1B). In addition, γδT + ATB had a more powerful therapeutic effect than γδT therapy alone (*p* = 0.013); statistics on the total fluorescence intensity from *in vivo* imaging also supported the same conclusion (Figures 1D,E). We also monitored the weight of these mice and found that there was no large difference among these four groups during the observation period (Figure 1C). Survival curves showed that γδT + ATB mice had a longer median survival time than the other three groups (Figure 1F). From the above phenomenon, we conclude that antibiotic treatment enhanced γδT cell efficacy during tumor immunotherapy in a mouse model.

### ATB Treatment Results in a Distinct Microbiota Distribution in the Gut of Tumor-Bearing Mice

**Figure 2:**
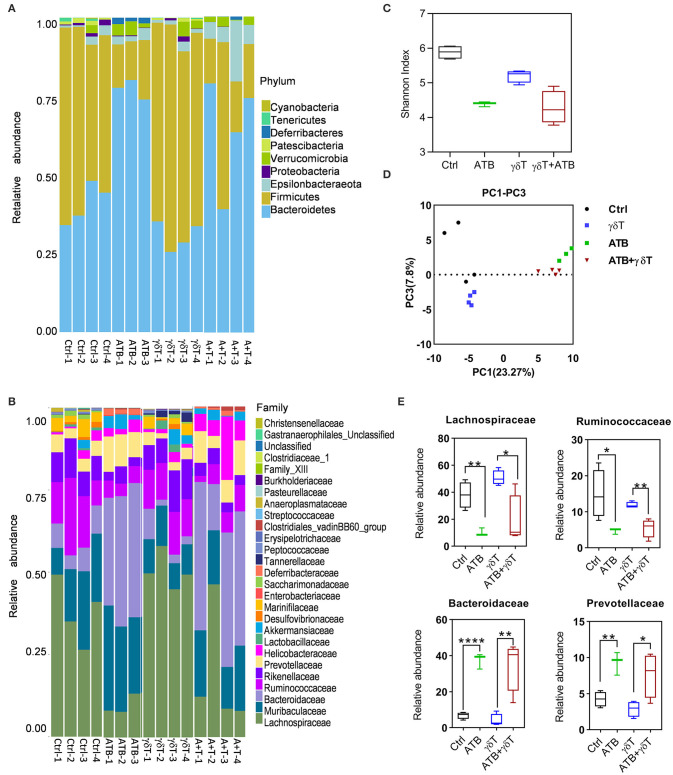
ATB treatment also changed the gut microbiota in tumor-bearing nude mice. **(A)** Relative abundance of the microbiota in the cecum of tumor-bearing mice at the phylum level. The columns represent the relative abundance of each sample, and the colors indicate the phylum name of the microbiota. **(B)** More details on the relative abundance of microbiota in the cecum at the family level. **(C)** α Diversity of samples in **(A)**; the *X*-axis represents group information, and the *Y*-axis represents the Shannon index. The higher the Shannon index is, the higher the diversity is. **(D)** Principal coordinate analysis shows the diversity of the samples in **(A)**; the *X*-axis represents the sample distribution in PC1, and the *Y*-axis represents the sample distribution in PC2. The greater the distance between two points is, the more different the abundance and diversity of the species contained in the two samples is. **(E)** Relative abundance of the four most significant families among these four groups of mice. Student's *t*-test. *p < 0.05, **p < 0.01, and ****p < 0.0001.

Considering that nude mice have different gut microbiota distributions compared to other mice and to determine the microbiome distribution caused by antibiotic treatment in tumor-bearing nude mice subjected to γδT therapy and which kinds of bacteria may contribute to enhancing the effects of γδT cell immunotherapy for tumors after ATB treatment, we detected the abundance and diversity of microbiota in the cecum contents of all four groups of mice. As the results show at the phylum level, Bacteroidetes, Verrucomicrobia, and Deferribacteres were more abundant, but Firmicutes was less abundant in the antibiotic-treated groups (ATB) than in the other three groups. In addition, the ATB combined with γδT therapy group had more Epsilonbacteraeota than the other groups (Figure 2A). Relative abundance analysis revealed that a substantial decrease in the abundance of the Lachnospiraceae and Ruminococcaceae families and an increase in the abundance of Bacteroidaceae and Prevotellaceae occurred in mice receiving ATB and γδT cell therapy (Figure 2B). Further α and β diversity analysis was performed to explore differences among these four groups and determine individual values for each group; it was obvious that ATB-treated groups had a lower Shannon index than the other three groups, which means that the cecum contents of these two groups have less community richness and diversity (Figures 2C,D). To better compare the bacterial distribution between mice treated with or without ATB, we identified the four families with the greatest differences among these four groups; the mice treated with ATB had more abundant Bacteroidaceae and Prevotellaceae but less Lachnospiraceae and Ruminococcaceae, ignoring with or without γδT cell therapy (Figure 2E).

From 16S sequencing, we found that the bacterial distribution in cecum feces from ATB-treated mice is different from that in cecum feces from non-ATB-treated mice; even γδT cell therapy alone can slightly change this distribution but is not as effective as ATB, which means that various treatments induce differences in the bacterial components of mouse feces, and these differences somehow lead to increased therapeutic efficacy of γδT cells against tumors. Therefore, we tried to find the mechanism underlying this effect.

### Cecum Contents From ATB-Treated Mice Enhance the Cytotoxicity of γδT Cells *in vitro*

**Figure 3:**
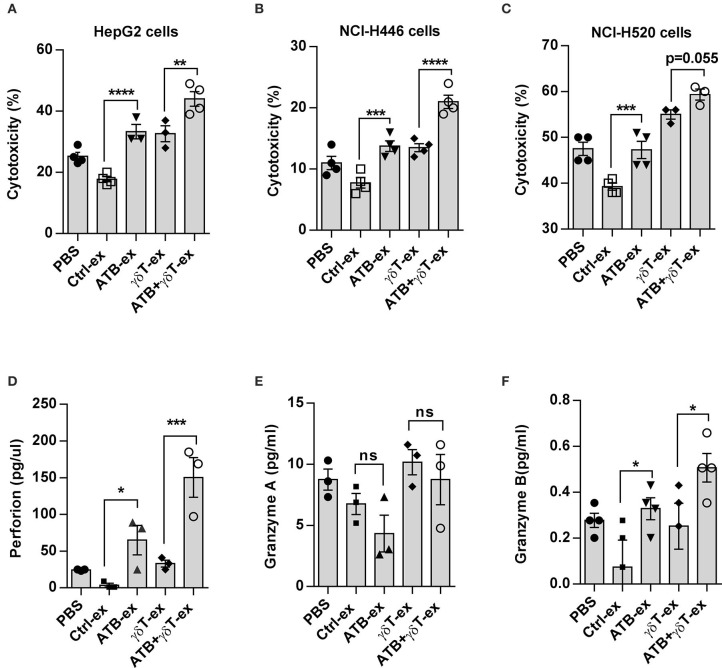
Cecum contents from ATB-treated mice enhance the cytotoxicity of γδT cells *in vitro*. **(A)** *In vitro* cytotoxicity of γδT cells against HepG2 hepatocellular carcinoma cells. Fecal extracts (ex) from different groups of mice or an equal volume of PBS were added into a γδT cell and HepG2 co-culture system for 4 h. Here, the results of four experiments are shown. Analysis of variance (ANOVA), **p < 0.01, and ***p < 0.001, ****p < 0.0001. **(B)** The same methods described in **(A)** were used to detect the cytotoxicity of γδT cells against NCI-H446 tumor cells and NCI-H520 tumor cells **(C)**. **(D)** Perforin secreted in the supernatant of γδT cells cultured with fecal extracts or PBS. *p < 0.05 and ***p < 0.001 (ANOVA). **(E)** Granzyme A detected with the same method in **(D)**. ns, no significant. **(F)** Granzyme B detected with the same method in **(D)**. The data represent three independent experiments. *P < 0.05 and (ANOVA).

According to previous results, we realized that feces from different groups have various microbiota compositions and thus may have distinct characteristics. To verify whether feces with diverse characteristics influence the function of γδT cells directly *in vitro*, we collected mouse feces from these groups and made sterile suspensions, which were added to a co-culture system of γδT cells and tumor cell lines. We detected the cytotoxicity of γδT cells against HepG2 tumor cells, and the results proved that compared to control co-cultures with an equal volume of PBS, γδT cell cytotoxicity was slightly decreased with feces from the blank group mice, even though the difference was not significant. However, cytotoxicity was remarkably improved in groups treated with feces from ATB-treated mice. The groups treated with feces from ATB mice and γδT + ATB mice had much higher cytotoxicity for tumor cells than did the groups treated with feces from control mice. Interestingly, compared to that of the PBS and blank control groups, γδT cell cytotoxicity was also increased in the group treated with γδT cell therapy alone (Figure 3A). We also conducted experiments with two other tumor cell lines, NCI-H520 and NCI-H446, and obtained consistent results (Figures 3B,C).

To determine how these intestinal content extracts increase the cytotoxicity of γδT cells for tumor cells *in vitro*, we detected cytotoxic cytokines secreted by γδT cells using enzyme-linked immunosorbent assay (ELISA). Pure γδT cells were cultured with fecal suspensions from different groups, and the supernatant was collected after 48 h. The results showed that γδT cells stimulated with feces from mice treated with either ATB or γδT cell therapy alone secreted less granzyme B and perforin than did cells stimulated with feces from mice treated with ATB combined with γδT cell therapy, but these cells still secreted more of these factors than did cells stimulated with feces from mice in the blank control group (Figures 3D,E); however, granzyme A secretion showed no difference among these groups (Figure 3F). These results indicate that feces from antibiotic-treated mice have the ability to alter the cytotoxicity of γδT cells *in vitro* by stimulating the secretion of the cytotoxic cytokines granzyme B and perforin.

### Cecum Contents From ATB-Treated Mice Do Not Contribute to the Proliferation of γδT Cells

**Figure 4:**
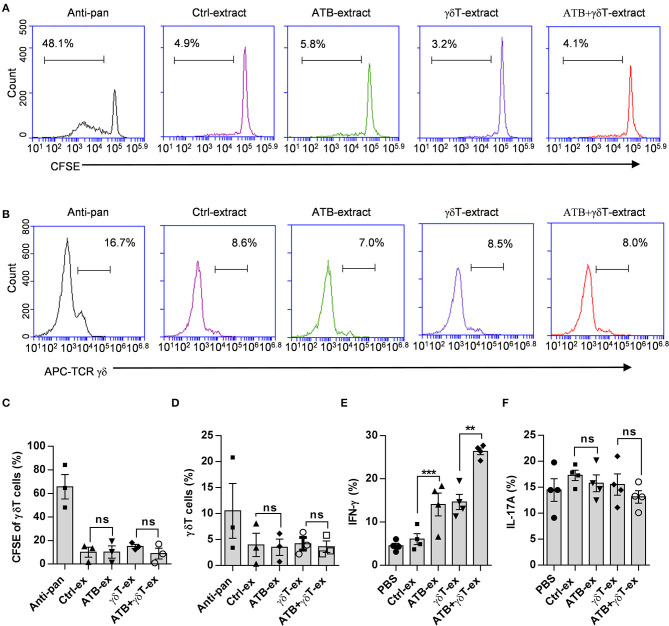
Cecum contents from ATB-treated mice do not contribute to the proliferation of γδT cells. **(A)** Representative plot of γδT cell proliferation. Peripheral blood mononuclear cells (PBMCs) isolated from healthy donors were stained with carboxyfluorescein succinimidyl ester (CFSE) and cultured in wells coated with anti-pan γδTCR or treated with fecal dilutions from different mice. **(B)** Representative plot of γδT cell frequencies in wells under the same conditions described in **(A)**. **(C)** Statistical diagram based on three experiments of γδT cell proliferation in **(A)**, ns, no significant. **(D)** Statistical charts of γδT cell purity based on three experiments of **(B)**. ns, no significant. **(E)** Interferon gamma (IFN-γ) secreted in the supernatant of γδT cells was detected using the same methods as described in Figure 3D. Student's *t*-test. **p < 0.01, ***p < 0.001. **(F)** Interleukin (IL)-17A secreted in the supernatant of γδT cells was detected using the same methods as described in Figure 3D, and the data represent three independent experiments, ns, no significant (ANOVA).

To confirm whether these cecum contents also influence the proliferation of γδT cells, we isolated PBMCs from healthy donors and stained them with carboxyfluorescein succinimidyl ester (CFSE). As a positive control, an anti-TCR pan-γδ antibody was used to effectively amplify human γδT cells, and untreated PBMCs were used as a negative control. Prepared fecal suspensions were added to each group, and after 5 days of culture, the purity and proliferation of γδT cells were detected by flow cytometry. As shown in Figure 4, there was a moderate increase in γδT cell purity and proliferation compared to the PBMC control, but no clear difference was observed among the groups (Figures 4A–D), which means that these fecal suspensions with different compositions cannot stimulate γδT cell proliferation. We next examined two important cytokines produced by γδT cells after stimulation with these fecal suspensions and found that feces from ATB-treated mice induced more IFN-γ production than feces from the other groups (Figure 4E), but this pattern was not observed for IL-17 (Figure 4F).

### More 3-Indolepropionic Acid Is Produced in Mice Treated With Antibiotics Combined With γδT Cell Therapy

**Figure 5:**
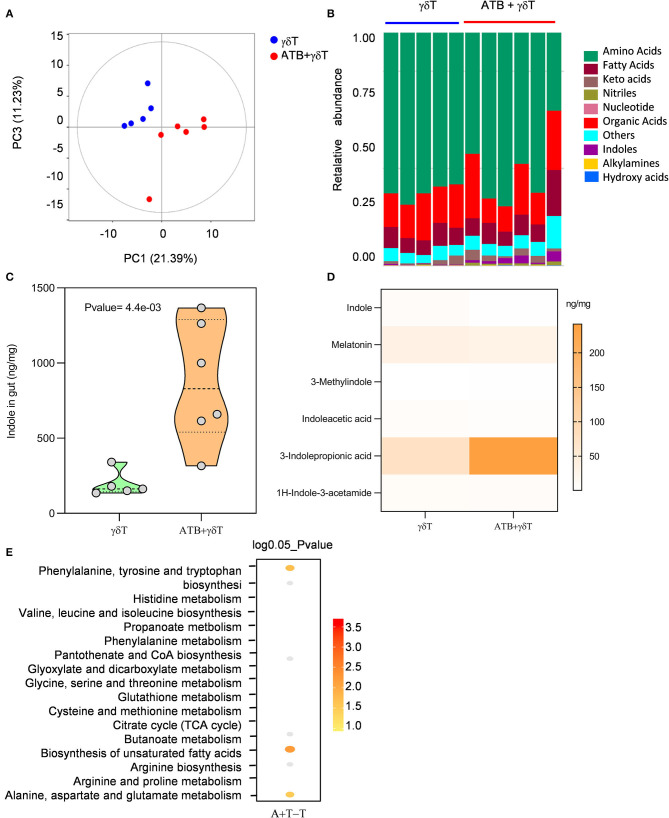
More 3-indolepropionic acid is produced in mice treated with antibiotics combined with γδT cell therapy. **(A)** Metabolites were detected in the cecum content by gas chromatography/mass spectrometry. Principal coordinate analysis shows the distribution of the samples in two principal components. The distance between the sample points represents the similarity of the metabolite communities in the sample; the closer the distance, the higher the similarity. **(B)** Relative abundance of metabolites in the same samples as **(A)**. **(C)** Concentration of indoles in samples from the γδT cell therapy group (*n* = 5) and the antibiotic combined with γδT cell therapy group (*n* = 6). Student's *t*-test. **(D)** Concentration heatmap of all listed indoles in the two groups. **(E)** MPEA analysis to predict which pathway contributes to the metabolic differences between the γδT cell therapy mice and the antibiotic-treated mice. The dot position on the *Y*-axis refers to a metabolic pathway; the color scale indicates the *p*-value, with darker dots indicating stronger correlations with the corresponding pathway.

Metabolite production is a critical factor by which the microbiota can regulate different systems. These metabolites circulate in the blood and then interact with local tissues (28–30). From previous results, it was already known that the microbiotas of all mice treated with antibiotics are similar in distribution and properties, regardless of whether they are treated with or without γδT cell therapy. Therefore, we focused on mice receiving γδT cell therapy with or without ATB and then tested metabolites related to the gut microflora by gas chromatography/mass spectrometry to explore the molecular mechanism by which ATB treatment leads to a better therapeutic effect of γδT cells. According to PCA analysis, compared to non-ATB treatment, ATB treatment led to a completely different metabolite composition (Figure 5A). Metabolite classes were detected, and we were surprised to find that more indoles were generated in mice treated with ATB than in mice treated with γδT cell therapy without ATB (Figure 5B). Further statistical analysis showed that the concentration of indoles was significantly increased in ATB-treated mice (Figure 5C). To investigate this issue in more detail, we measured different kinds of indoles and observed that 3-indolepropionic acid (IPA) was substantially more abundant than the other indoles in ATB-treated mice (Figure 5D). Metabolite pathway enrichment analysis (MPEA) is a widely used approach to reveal altered pathways among diverse samples based on GC-MS data (29). We performed MPEA according to metabolite profiles to determine which metabolic pathways are altered in mice treated with antibiotics combined with γδT cell therapy compared to mice treated with γδT cell therapy alone. Our results revealed that three pathways could be changed after antibiotic treatment: phenylalanine, tyrosine, and tryptophan metabolism; biosynthesis of unsaturated fatty acids; and alanine, aspartate, and glutamate metabolism (Figure 5E).

### 3-Indolepropionic Acid Enhances the Cytotoxicity of γδT Cells Both *in vitro* and *in vivo*

**Figure 6:**
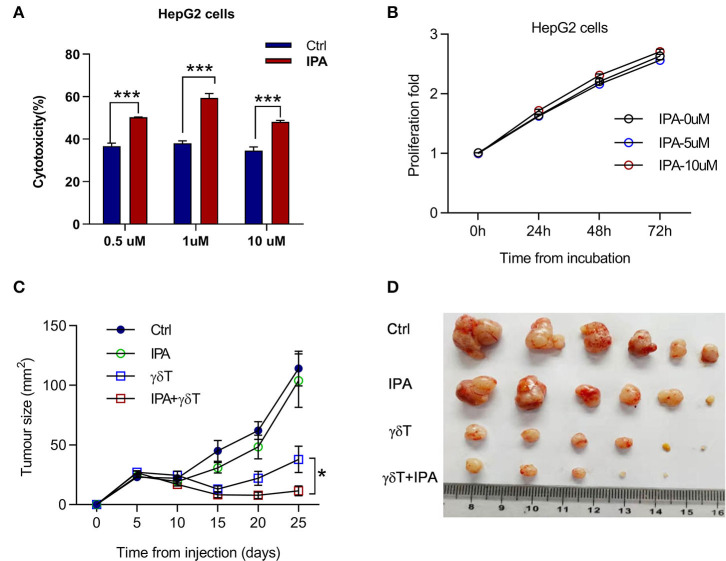
3-Indolepropionic acid enhances the cytotoxicity of γδT cells both *in vitro* and *in vivo*. **(A)** 3-Indopropionic acid (IPA) or an equal volume of phosphate-buffered saline (PBS) was added to a γδT cell and HepG2 co-culture system for 4 h, and the cytotoxicity of γδT cells was detected by using a Cytototo96 kit. The plot shows four independent experiments. ***p < 0.001 (unpaired *t*-test). **(B)** HepG2 cells were cultured in 96-well plates for 72 h, 10,000 cells per well and 5 μM IPA, 10 μM IPA, or an equal volume of medium were added to examine proliferation and viability by MTT (cell proliferation kit 1). Proliferation was calculated as the measured value/original value. **(C)** Tumor growth of HepG2-bearing nude mice treated with or without (PBS control) IPA three times or IPA combined with γδT cell therapy. Data are the mean ± SEM of the tumor sizes for six mice in each group. *p < 0.05 (ANOVA). **(D)** Image of the tumor from the mice in **(C)**.

In summary, we propose that IPA may play a crucial role in the change in γδT cell efficacy for tumors under antibiotic treatment. We added different doses of IPA to the tumor cells and γδT cell co-culture system, and the cytotoxicity of γδT cells was examined after 4 h. An equivalent solvent was added to the control group. Interestingly, we found that the cytotoxicity of γδT cells was obviously improved by IPA (Figure 6A). To explore whether IPA causes damage to tumor cells directly in this system at the doses we used, we monitored the viability of tumor cells treated with different doses of IPA. The data revealed no significant damage to tumor cells after culture with IPA, especially during the first 24 h (Figure 6B). Next, to test whether IPA also regulates γδT cell cytotoxicity *in vivo*, we examined the tumor size of tumor-bearing nude mice under γδT cell therapy with or without IPA. Consistent with the *in vitro* results, IPA treatment also promoted the therapeutic efficacy of γδT cells (Figures 6C,D).

## Discussion

Increasing evidence has shown that the microbiota plays an important role in the process of cancer immunotherapy. People with different dominant gut microbiota have distinct sensitivity to immunotherapy. Patients with abundant *Akkermansia muciniphila* in the stool respond to antiprogrammed cell death 1 protein (PD-1)-based immunotherapy well (24). Melanoma patients with a higher relative abundance and alpha diversity of the Ruminococcaceae family respond to PD-1 therapy better (23). Except for these special bacteria, as a study shows, the overall abundance of microbiota influences tumor development as well. Decreasing the quantity of bacteria in antibiotic-treated or germ-free mice resulted in protection from lung cancer. Additionally, the products generated by microbiomes, the crosstalk between them and the immune cells are important mechanisms by which bacteria influence tumor growth (26, 31).

In this study, we found that antibiotic treatment decreases the frequency of γδT and NK cells in the spleen while increasing both the frequency and cell number of IEL γδT cells. The main expanded populations are Vγ1 and Vγ4, rather than Vγ1^−^Vγ4^−^ cells, which is the most distributed γδ subset in the small intestinal tract of mice. We know that ~90% of IEL γδT are CD8αα phenotype (32), the majority of them are Vγ1^−^Vγ4^−^ (Vγ7). It was reported that the maturation and proliferation of these cells are modulated by butyrophilin-like 1 (btnl1) molecules expressed in the gut epithelium, instead of being dependent on microbial or food antigens (33), which could be the reason why this population is not as sensitive as the Vγ1 and Vγ4 populations to antibiotic treatment.

Most importantly, we also found that antibiotic treatment improves the efficacy of γδT cells in tumor therapy, and gut microorganisms and their related metabolites are crucial factors underlying this phenomenon. From our model, the characteristics of the intestinal flora distribution in antibiotic-treated mice were completely different from those in normal mice; they are abundant with Bacteroidaceae, Muribaculaceae, and Prevotellaceae and lack Lachnospiraceae and Ruminococcaceae at the family level, regardless of whether they received γδT cell therapy. It also appears that γδT cell therapy causes subtle disorder of the gut microbiota, but the diversity and abundance of the microbiota are largely unaltered compared to normal mice. Although gut microbiota distribution was highly changed in antibiotic-treated mice during γδT cell immunotherapy, we are not focused on this point, as it is difficult to conclude which bacteria are the most important in this process, and it is still unclear how the gut microbiota interacts with the distant immune system. In our opinion, metabolites produced by bacteria might be messengers that connect gut microbiota and systemic immunity based on our results.

Cecum content that contains different bacteria has distinct properties. Content from antibiotic-treated mice has the property of enhancing the cytotoxicity of γδT cells *in vitro* by stimulating the increased secretion of cytotoxic cytokines, such as granzyme B and perforin. However, they have no effect on γδT cell proliferation and expansion but can boost the production of IFN-γ rather than IL-17. By performing GC-MS for metabolic profiling, we found that indoles are the most significant substance between antibiotic- and non-antibiotic-treated tumor-bearing mice when they received γδT cell therapy, and IPA is the highest concentration molecule in mice treated with antibiotics when combined with γδT cell therapy. We then found that IPA plays a role in enhancing γδT cell cytotoxicity, both *in vitro* and *in vivo*.

Based on previous studies, indoles originate from tryptophan metabolism, and bacteria that highly express tryptophanase can decompose tryptophan into indoles (34). IPA is an important metabolite produced in this way. Studies have shown that IPA is essential for the protection of primary neurons and neuroblastoma cells against oxidative damage caused by β-amyloid protein and H_2_O_2_ (35). It also has a beneficial function to protect people from diabetes or nervous system disease, and a high concentration of IPA in human plasma is related to a lower risk of type 2 diabetes and is beneficial for Alzheimer's disease (11, 36). Furthermore, high concentrations (10 mM) of IPA *in vitro* increase membrane fluidity, which is relevant to cancer prevention (37). The origin of IPA remains indistinct, and it has been reported that IPA is abundant in germ-free mice, as well as mice treated with broad-spectrum antibiotics, which suggests that the normal composition of the gut microbiota in mice may be disadvantageous to the generation of IPA and that when the quantity of bacteria is decreased by antibiotics, it can be produced more. Some studies have reported that IPA can only be detected in human serum when the bacterium *Clostridium sporogenes* is present in the gut (38–40); according to our results, *C. sporogenes* is not abundant in antibiotic-treated mice, despite the presence of high levels of IPA. One of the possible reasons is that there is some difference between humans and mice in terms of IPA origin, and the quantity of IPA could be the result of the activation of several bacteria, rather than depending on one bacterium or one pathway. These bacteria may cooperate together or restrain each other and eventually promote IPA production. In our study, phenylalanine, tyrosine, and tryptophan metabolism, the biosynthesis of unsaturated fatty acids, and alanine, aspartate, and glutamate metabolism are all possible pathways related to IPA enhancement, and numerous floras contribute to this process. As a ligand for the pregnane X receptor, IPA effectively activates the PXR receptor and downregulates the mRNA expression of TNF-α to protect the intestine from inflammation and maintain mucosal integrity (39).

As we mentioned, there are still several deficiencies in our study because of technology and theory problems. We cannot find specific bacteria that dominate IPA production from the above results, and it is difficult to prove which bacteria are the most valuable. On the other hand, we found that IPA is an important factor for enhancing γδT cell cytotoxicity in antibiotic-treated mice, and other metabolites may also play a role in this process. From our experiment, antibiotic treatment combined with γδT cell therapy was slightly more efficient than IPA treatment. Moreover, we did not find appropriate technology to track the process of IPA production and its crosstalk with γδT cells. Although no clear evidence so far has shown the exact mechanism by which gut microbiota regulate the antitumor effect of immune therapy, especially for specific immune cell therapy, we could infer based on published articles and our results that the diversity and distribution of bacteria in the gut are important to maintain homeostasis. Metabolites produced by these bacteria are the most likely mediators interacting with the remote immune system when the homeostasis of gut bacteria is disordered by antibiotics. We know that these metabolites circulate with blood and thus spread in other tissues. Therefore, we tend to believe that antibiotic treatment diminishes gut bacteria and somehow influences tryptophan metabolism, one of the sources of IPA production to be considered currently, thus increasing the concentration of IPA in the gut. They encounter injected γδT cells either in peripheral blood or in the tumor microenvironment and stimulate them to produce more cytotoxic cytokines, such as granzyme B and perforin, to destroy tumor cells. We will try to explore the above problems in further studies.

## Data Availability Statement

The raw data of 16S rRNA sequence in this study can be found here: https://www.ncbi.nlm.nih.gov/Traces/study/?acc=PRJNA612945.

## Ethics Statement

Ethical review and approval was not required for the study on human participants in accordance with the local legislation and institutional requirements. The patients/participants provided their written informed consent to participate in this study. This animal study was reviewed and approved by Animal Care and Use Committee of Peking Union Medical College.

## Author Contributions

JZ, WH, HC, JH, and WC: conceptualization. JH, YX, SZ, and YP: methodology and investigation. JZ, JH, and YP: writing—original draft. WC, HC, WH, and JZ: writing—review and editing. JZ and WH: funding acquisition and supervision. YH and HC: resources. All authors contributed to the article and approved the submitted version.

## Conflict of Interest

The authors declare that the research was conducted in the absence of any commercial or financial relationships that could be construed as a potential conflict of interest.
