# Supplementary material for: Beneficial Effect of Antibiotics and Microbial Metabolites on Expanded Vδ2Vγ9 T Cells in Hepatocellular Carcinoma Immunotherapy
Source: Front Immunol. 2020 Jul 22;11:1380. doi: 10.3389/fimmu.2020.01380 (PMC7396509; doi:10.3389/fimmu.2020.01380)
Supplement: Supplementary file 1 [file Data_Sheet_1.PDF]

Supplementary Material

Supplementary Figures

Supplementary Figure 1

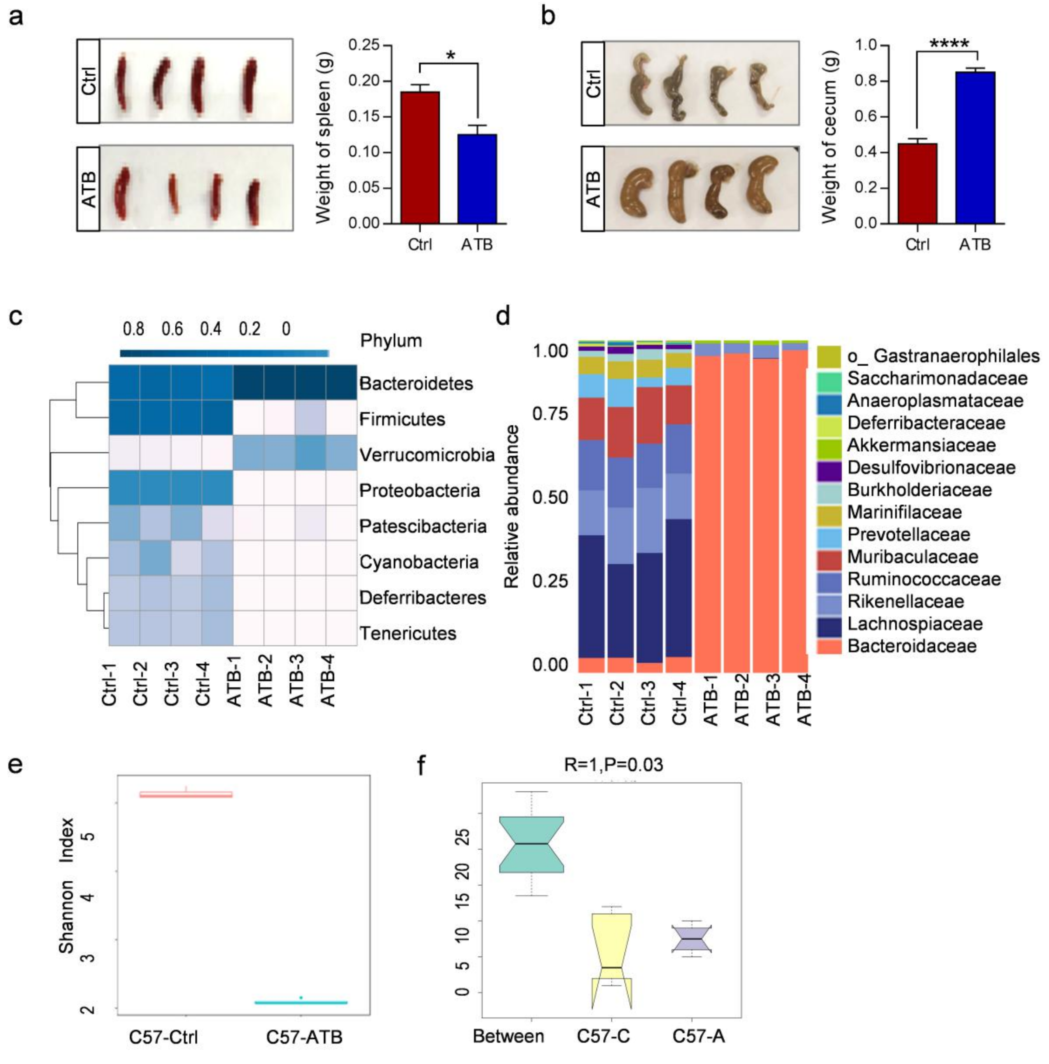

Supplemental Figure 1 Diversity and abundance of gut microbiota decreased in antibiotic treated mice**. (a)** Picture of spleen from C57BL/6J mice with or without antibiotic treatment. **(b)** Picture of cecum from same mice with a. **(c)** Heatmap of microbial abundance in same mice with a, the column is sample information and the row is the Phylum name of microbiota. **(d)** Relative abundance of microbiota in Family level, the column in different color represent different family and their proportion in this sample, relative abundance of each sample. The rainbow on the right labeled name of microbiota. **(e)** Shannon index, the higher Shannon index, the more abundant diversity. **(f)** Anosim

analysis to reveal microbiota difference between control and antibiotic treated group, the ordinate is the rank of the distance between samples, closer to 1 of R value, more big difference between two groups, P value equal 0.03, the difference is significant in statistics.

Supplemental Figure 2

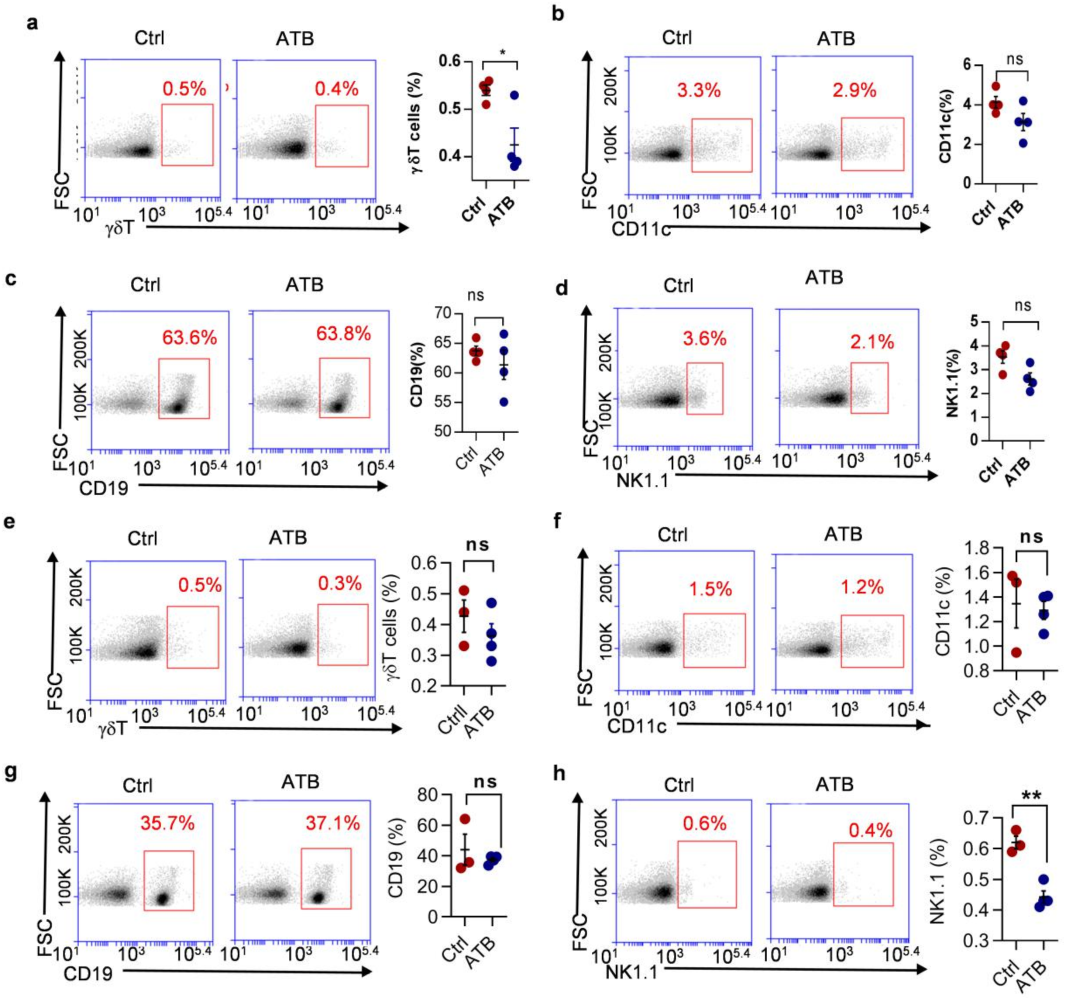

Supplemental Figure 2 Antibiotics treatment changes frequency of peripheral immune cells. **(a-d)** Staining of TCR γδ, CD11c, CD19 and NK1.1 in spleen of 8 weeks C57BL/6J mice with or without antibiotic treatment. **(e-h)** Staining of TCRγδ, CD11c, CD19 and NK1.1 in mesenteric lymph node of 8 weeks C57BL/6J mice with or without antibiotic treatment in same mice with **a**, *P < 0.05 and **P < 0.005, ns means P >0.05 (unpaired two-tailed Student's t-test). Data are representative of at least three independent experiments (mean and SEM).

Supplemental Figure 3

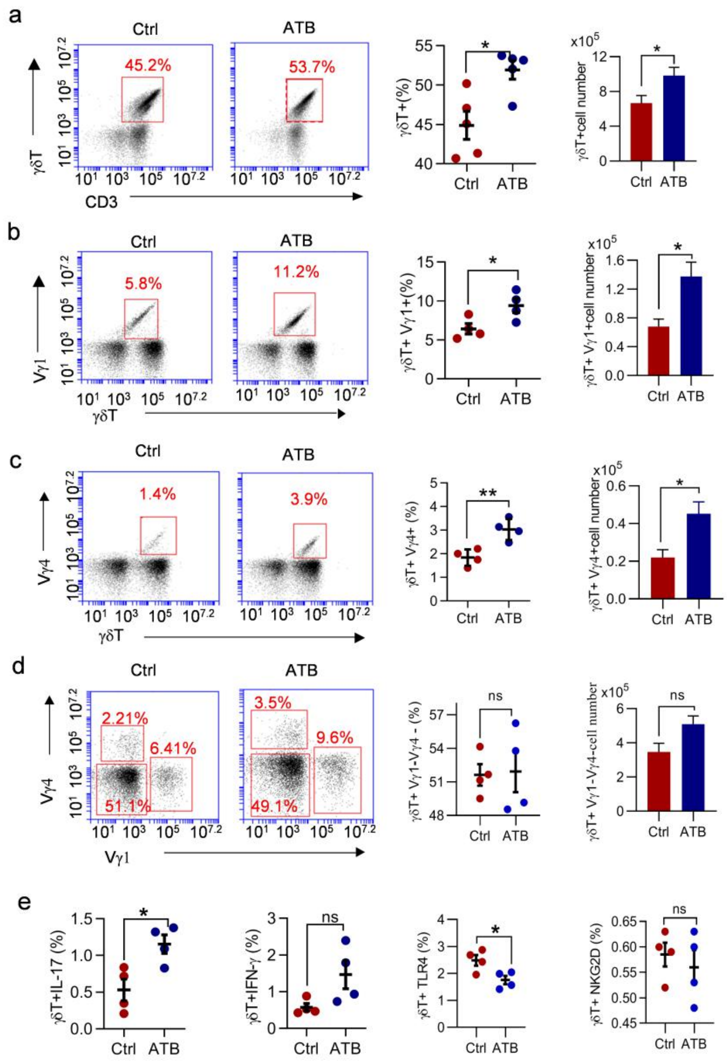

Supplemental Figure 3 Mice treated with antibiotics have more γδIELs. **(a)** Staining of CD3 and TCRγδ in IELs from 8-week-old C57BL/6J mice with or without antibiotic treatment, as well as frequency and total number. **(b)** Staining of Vγ1 and TCRγδ in the CD3+ gate and frequency and total number from the same mice in **a**. **(c)** Staining of Vγ4 and TCRγδ in the CD3+ gate and frequency and total number from the same mice in **a** (4 mice each group). **(d)** Staining of Vγ1 and Vγ4 in the CD3+ TCRγδ gate and frequency and total number from the same mice in **a**. **(e)** Frequency of IL-17, IFN-γ, TLR4 and NKG2D in the CD3+ TCRγδ gate. *P < 0.05 and **P < 0.005, ns means not significant, P >0.05 (unpaired two-tailed Student's t-test). Data are representative of at least three independent experiments (mean and SEM).

Supplemental Figure 4

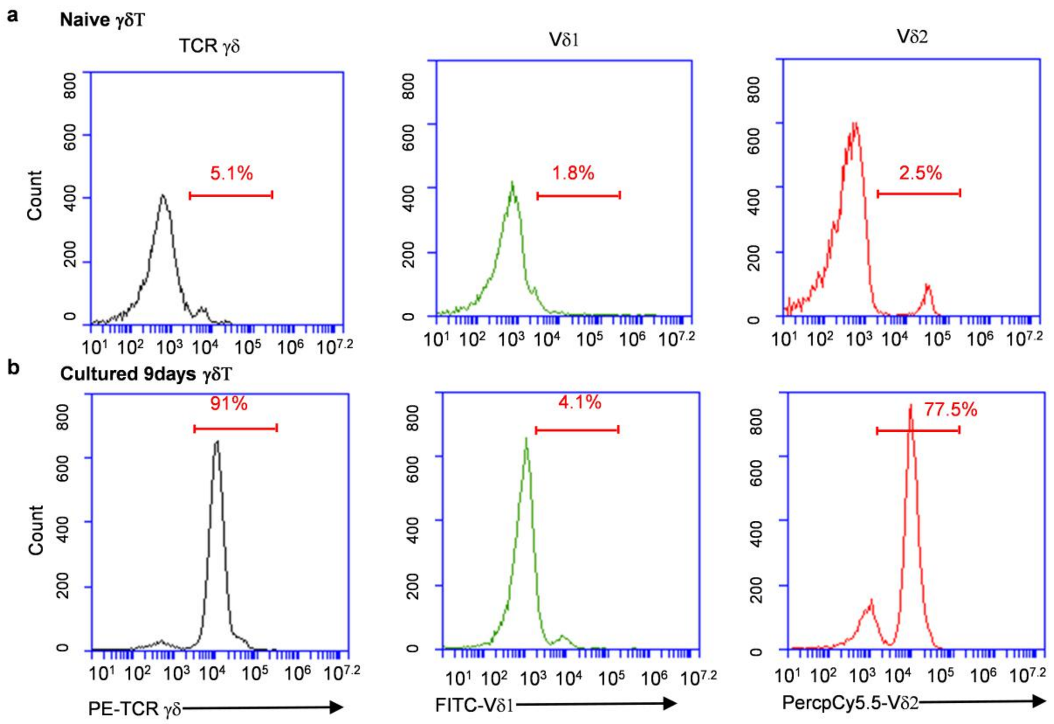

Supplemental Figure 4 Human peripheral blood γδT cells were expended with TCR pan-γδ antibody. **(a)** Representative plots of frequency for naïve TCRγδ and its' subsets Vδ1 and Vδ2 in human peripheral blood mononuclear cells. **(b)** PBMC were suspended in complete RPMI-1640 medium and cultured in 24 well plate that coated with 10ug TCR pan-γδ 37°C 2 hours, 2×10^6 cells per well. 4 days later, transfer the cells into new well to eliminate the stimulation and continue to culture, on the 9th day culture, the purity of TCRγδ, Vδ1 and Vδ2 are as above plot showing.

Methods and materials

Animal models 8-week-old adult C57BL/6J black mice were housed in the Experimental Animal Center of Peking Union Medical College under specific-pathogen-free (SPF) conditions. All of the procedures in this study were authorized and supervised by the Animal Care and Use Committee of Peking Union Medical College.

Antibiotic treatment C57BL/6J mice were given drinking water containing antibiotics (penicillin 0.8 mg/ml, chloramphenicol 1 mg/ml, streptomycin 1.2 mg/ml)(Gopalakrishnan et al., 2018; Routy et al., 2018; Jin et al., 2019) or normal water one week, then took spleen, gut and mesenteric lymph node.

16S rRNA gene sequencing DNA was extracted from fecal samples, and the concentration was detected using the Qubit® dsDNA HS Assay Kit. Then, the highly variable region (V3 and V4) of 16S rDNA was amplified with primers designed by GENEWIZ. Concentrations were then measured with a Qubit3.0 Fluorometer (Invitrogen, Carlsbad, CA) and adjusted to 10nM for PE250/FE300 double-end sequencing. Finally, data analysis was performed.

Flow cytometry and data analysis Single cells from mouse or cultured human PBMC were washed with PBS, then staining antibody at 4°C, 30minutes after blocking non-specific bonding site on cells with 1% BSA buffer. Washing samples 2 times to remove excess antibody then test samples on BD Accuri™ C6 Cytometer and analysis data by BD Accuri™ C6 Cytometer analysis software.

IEL isolation Small intestine was isolated from mice, Peyer's patches and fat attached to the tissue were removed, and the intestine was cut open. Feces and mucus were washed away with PBS, and the gut tissue was cut into pieces, suspended in RPMI-1640 plus 5% fetal bovine serum and placed on a shaker for 20 min (37°C, 180 rpm). Then, the samples were centrifuged at 300g for 10 min, the cells were suspended in 44% Percoll, and 70% Percoll was added slowly from the bottom. The samples were centrifuged at 1800 rpm for 20 min with brake 0, and lymphocytes were obtained between these two layers.(Konkel et al., 2011; Di Marco Barros et al., 2016)

Human PBMC isolation and γδT cell expansion Fresh human PBMCs were separated from peripheral blood from healthy donors by density gradient centrifugation on Ficoll-Hypaque (Pharmacia) and cultured using RPMI-1640 (Gibco BRL) medium with 10% FCS and IL-2 (200 U/ml) (Giri et al., 1994; Hintz et al., 2001) in 24-well culture plates coated with an anti-TCR pan-γδ antibody (2 ng/ml)(Kondo et al., 2011) at a density of 2×10^6 cells per well to expand pure γδT cells.

Reference

Di Marco Barros, R., Roberts, N.A., Dart, R.J., Vantourout, P., Jandke, A., Nussbaumer, O., et al. (2016). "Epithelia Use Butyrophilin-like Molecules to Shape Organ-Specific gammadelta T Cell Compartments", in: *Cell.*).

Giri, J.G., Ahdieh, M., Eisenman, J., Shanebeck, K., Grabstein, K., Kumaki, S., et al. (1994). Utilization of the beta and gamma chains of the IL-2 receptor by the novel cytokine IL-15. *EMBO J* 13(12)**,** 2822-2830.

Gopalakrishnan, V., Spencer, C.N., Nezi, L., Reuben, A., Andrews, M.C., Karpinets, T.V., et al. (2018). Gut microbiome modulates response to anti-PD-1 immunotherapy in melanoma patients. *Science* 359(6371)**,** 97-103. doi: 10.1126/science.aan4236.

Hintz, M., Reichenberg, A., Altincicek, B., Bahr, U., Gschwind, R.M., Kollas, A.K., et al. (2001). Identification of (E)-4-hydroxy-3-methyl-but-2-enyl pyrophosphate as a major activator for human gammadelta T cells in Escherichia coli. *FEBS Lett* 509(2)**,** 317-322. doi: 10.1016/s0014-5793(01)03191-x.

Jin, C., Lagoudas, G.K., Zhao, C., Bullman, S., Bhutkar, A., Hu, B., et al. (2019). Commensal Microbiota Promote Lung Cancer Development via gammadelta T Cells. *Cell* 176(5)**,** 998-1013 e1016. doi: 10.1016/j.cell.2018.12.040.

Kondo, M., Izumi, T., Fujieda, N., Kondo, A., Morishita, T., Matsushita, H., et al. (2011). Expansion of human peripheral blood gammadelta T cells using zoledronate. *J Vis Exp* (55). doi: 10.3791/3182.

Konkel, J.E., Maruyama, T., Carpenter, A.C., Xiong, Y., Zamarron, B.F., Hall, B.E., et al. (2011). Control of the development of CD8alphaalpha+ intestinal intraepithelial lymphocytes by TGF-beta. *Nat Immunol* 12(4)**,** 312-319. doi: 10.1038/ni.1997.

Routy, B., Le Chatelier, E., Derosa, L., Duong, C.P.M., Alou, M.T., Daillere, R., et al. (2018). Gut microbiome influences efficacy of PD-1-based immunotherapy against epithelial tumors. *Science* 359(6371)**,** 91-+. doi: 10.1126/science.aan3706.
